# Vergleichsverbot? Bevölkerungsstatistiken und die Frage der Vergleichbarkeit in den deutschen Kolonien (1885–1914)

**DOI:** 10.1007/s11577-021-00745-z

**Published:** 2021-05-06

**Authors:** Léa Renard

**Affiliations:** ZI Lateinamerika-Institut, Freie Universität Berlin, Boltzmannstr. 1, 14195 Berlin, Deutschland

**Keywords:** Klassifikation, Kolonialismus, Zensus, Territorialität, Deutsches Kaiserreich, Classification, Colonialism, Census, Territoriality, Empire

## Abstract

Der Beitrag untersucht das Verhältnis von statistischen Klassifikationen und Vergleichen in deutschen Kolonialstatistiken zwischen 1885 und 1914 und geht der Frage nach, welche Bedeutung und welche Ausprägungen dem Vergleich in Bezug auf Raum und Bevölkerung in kolonialen Statistiken zukamen. Ziel ist, den Blick für Methoden und Kategorien der statistischen Praxis im imperialen Kontext zu schärfen. Die Ergebnisse zeigen, dass der statistischen Beobachtung der Kolonien eine territoriale Grundunterscheidung zwischen Metropole und Kolonien zugrunde lag, die in unterschiedlichen Methoden mündete. Diese territoriale und methodologische Spaltung war mit einer grundsätzlichen Unvergleichbarkeit zwischen kolonisierter Bevölkerung und Kolonisierenden gekoppelt, so die These des Beitrags.

## Abstract (en)

This article explores the relationship between statistical classifications and comparisons in German colonial statistics between 1885 and 1914. It questions the importance and the characteristics of comparison in terms of space and population in colonial statistics. The aim is to sharpen the view of statistical methods and categories in an imperial context. The results show that the statistical observation of colonies was based on a territorial distinction between metropole and colonies, which led to the use of different methods. I argue that this territorial and methodological distinction was interwoven with a fundamental incomparability between colonized populations and colonizers.

## Einleitung

Als zwei Formen moderner Staatlichkeit waren Empires und Nationalstaaten im 19. Jahrhundert stark ineinander verwoben. Historische Studien weisen einerseits auf die Nationalisierung von Empires und andererseits auf imperiale Elemente in Nationalstaaten hin (Burbank und Cooper 2010; Leonhard und von Hirschhausen 2011, S. 107 ff.). In den letzten Jahren werden zunehmend auch Soziologinnen und Soziologen – insbesondere im Bereich der globalen historischen Soziologie – dazu ermutigt, das Empire als die bis in die zweite Hälfte des 20. Jahrhunderts „dominant sociopolitical formation of modernity“ ernst zu nehmen (Go 2019; s. a. Wimmer und Min 2006; Rapior 2020). Das Empire als politisches Ordnungsmodell war durch die Bestrebung gekennzeichnet, heterogene Gruppen und diverse Räume durch unterschiedliche Methoden zu verwalten, die auch als „Politik der Differenz“ beschrieben worden sind (Burbank und Cooper 2010).^1^ Umso erstaunlicher ist es, dass die Soziologie und die Geschichte der Statistik staatliche Quantifizierungspraktiken bislang vorwiegend bezogen auf das Aufkommen des Nationalstaates untersucht haben und imperialen Statistiken kaum Aufmerksamkeit geschenkt wurde.^2^ Im deutschen Fall kommt hinzu, dass das Kaiserreich in der Geschichtsschreibung bis vor rund zwei Jahrzehnten selten unter dem Aspekt seines Kolonialismus analysiert wurde (Conrad und Osterhammel 2004). Auch für die Vergleichsforschung sind koloniale Statistiken ein bislang unerforschtes Feld: Jüngere historische und soziologische Studien haben zwar die Bedeutung des quantifizierten Vergleichs in Globalisierungsprozessen hervorgehoben (Heintz und Werron 2011; Renard und Wobbe 2019), die Rolle des statistischen Vergleichs als koloniale Technologie im Prozess des Empire-Building wurde dagegen noch nicht hinreichend analysiert. Im weltpolitischen Kontext des 19. Jahrhunderts diente die Statistik als zwischenstaatliche Konvention zur wechselseitigen Beobachtung und (Ver‑)Messung (Speich Chassé 2020), wie dies auf den internationalen statistischen Kongressen (1853–1876) und ab 1885 im Rahmen des International Statistical Institute diskutiert wurde (Brian 1989; Randeraad 2010). Im Zusammenhang mit dem internationalen Wettbewerb um Territorien und ihre ökonomischen Ressourcen waren Statistiken eine gemeinsame Sprache, die es ermöglichte, Empires zu vergleichen (dazu auch Steinmetz 2021).

Der Beitrag untersucht daher das Verhältnis von statistischen Klassifikationen und Vergleichen in deutschen Kolonialstatistiken^3^ in Bezug auf die Vermessung der Bevölkerung zwischen 1885 und 1914. Die Entstehung der Bevölkerung als ein abstraktes Konzept ist stark mit der Geschichte der modernen Herrschaftsform verwoben, die Michel Foucault (2004) als „Gouvernementalität“ bezeichnet hat (vgl. auch Gutiérrez Rodriguez und Pieper 2003). In seiner Vorlesung „Sicherheit, Territorium und Bevölkerung“ (1977–1978) beschreibt er für das 19. Jahrhundert die Entstehung einer neuen Form des Regierens, die auf die *Reg(ul)ierung* der Bevölkerung als eigenständiger Entität mit eigenen Regeln und Dynamiken zielt. Statistische Methoden und demografische Konzepte, wie Mortalität und Natalität, seien paradigmatisch für diese Regierung des Lebens, die er als „Biopolitik“ bezeichnet.^4^ Während Foucault selbst seine Analysen auf die (nationale) Bevölkerung beschränkte, haben zahlreiche Autorinnen und Autoren^5^ die Fruchtbarkeit seiner Theorie hervorgehoben, um auch über moderne Räumlichkeit und insbesondere koloniale Raumordnungen nachzudenken (Kalpagam 2014, S. 83 ff.; Crampton und Elden 2007).^6^ Mit Alain Desrosières (1997) gehe ich davon aus, dass mit der Praxis des Zensus^7^ um 1900 nicht nur ein abstraktes Bevölkerungskonzept, sondern auch ein bestimmtes Verhältnis zum Territorium verbunden ist. Ein grundlegender Unterschied im Vergleich zu neuen digitalen Beobachtungsformen, die als ortsungebundene Verfahren geografische Distanzen und Kopräsenz überwinden (Heintz 2021; Harbach 2012), liegt in der territorialen Verankerung der Statistik um 1900. Vor dem Hintergrund dieser Diskussion lässt sich fragen, welche Bedeutung und welche Ausprägungen dem Vergleich in Bezug auf Bevölkerung und Raum in kolonialen Statistiken zukamen.

Vergleiche stellen Zusammenhänge zwischen Gegenständen, Phänomenen, Personen, Gruppen oder gar Staaten her, die zuvor unter Umständen voneinander getrennt wahrgenommen wurden. Sie sind Techniken zur Wissensgenerierung und damit soziale Praktiken (vgl. Epple und Erhart 2015; Steinmetz 2019). Um Einheiten miteinander vergleichen zu können, müssen diese in mindestens einer Hinsicht als gleichartig angesehen werden, bevor sich Ähnlichkeiten oder Unterschiede anhand von ausgewählten Kriterien feststellen lassen (Heintz 2016, S. 307). Dies setzt voraus, dass die verglichenen Entitäten zunächst derselben übergeordneten Kategorie (in diesem Fall dem Konstrukt „Bevölkerung“) zugerechnet werden. Der Vergleich wird somit durch „Äquivalenzkonventionen“ ermöglicht (Desrosières 2007, S. 199; vgl. auch Desrosières 2001). Denn was und wer in einer Gesellschaft als gleichartig oder äquivalent gilt, ist hochgradig kontingent und von der Sozialstruktur abhängig. „Vergleichen (das heißt *zusammen betrachten*) ist ein politischer Akt“ (Desrosières 2007, S. 174, eigene Übersetzung). Bezogen auf soziale Gruppen kann das dazu führen, dass „in einigen Gesellschaften Sklaven und Freie, Frauen und Männer, Nichtadelige und Adelige, Schwarze und Weiße nicht verglichen werden konnten (im Sinne von ‚es war nicht denkbar …‘)“ (Desrosières 2007, S. 174, eigene Übersetzung). Wenn ein Vergleich aufgrund der geltenden Normen oder Vorstellungen als nicht zulässig erklärt wird, so besteht ein Vergleichsverbot (Heintz 2016, S. 307).

Die Herausbildung statistischer Klassifikationssysteme, die Auswahl von Ordnungsprinzipien, die Formierung einzelner Subkategorien sowie die Zuordnung konkreter Entitäten zu abstrakten Klassen sind voraussetzungsreiche Operationen, die auf sozialen Aushandlungsprozessen und oftmals selbst auf Vergleichen beruhen (Douglas 1986). Wenn Entitäten zusammen in eine Kategorie gebracht werden, dann betont die kategorisierende Instanz ihre Gemeinsamkeiten, während die Unterschiede in den Hintergrund treten – etwa Klassenunterschiede innerhalb der Kategorie „Frau“ (Zerubavel 1996). Insofern sind Kategorisierung und Vergleiche in konkreten Situationen, wie z. B. in der statistischen Praxis, oft miteinander verstrickt.

Als Materialgrundlage für die empirische Analyse von deutschen Kolonialstatistiken dienen hier die von der Kolonialverwaltung zwischen 1885 und 1914 (ab 1892 jährlich) veröffentlichten „Denkschriften über die deutschen Schutzgebiete“^8^ (ausführlicher Renard 2021). Ein zeitgenössischer Beobachter, der bayerische Bezirksamtsassessor Rudolf Hermann (1911, S. 940), beschrieb einst die Kolonialstatistik als „Fremdkörper“, der sowohl bezüglich des Gegenstands als auch der Methode einen Gegenpol zur „Reichsstatistik“ in der Metropole bilden würde. In der Tat oblag die Aufbereitung statistischen Materials für die Kolonien nicht dem Statistischen Reichsamt, sondern der Kolonialverwaltung. Welche Konsequenzen sich für die Datenerfassung und Kategorisierung der Bevölkerung daraus ergaben, ist Gegenstand der nächsten Teile. Die Denkschriften bezogen sich auf die deutschen Kolonien in Afrika (Togo, Kamerun, „Deutsch-Ostafrika“^9^, „Deutsch-Südwestafrika“^10^) und im Pazifik (Samoa, „Deutsch-Neuguinea“^11^).^12^ Darüber hinaus wurde der Schriftverkehr um die Redaktion der Denkschriften in den Akten des Reichskolonialamts, die im Bundesarchiv konserviert und digital zugänglich sind, systematisch untersucht. Beleuchtet werden hiermit Praktiken des Vergleichs im Spannungsverhältnis der wechselseitigen Konstitution von politischem Ordnungsmodell und Gesellschaft, um so den Blick für Methoden des Zählens und deren historische Kontextbedingungen zu schärfen.

Der erste Teil skizziert die Produktion von Bevölkerungsbeobachtungen zum Zweck der Kolonialpolitik in verschiedenen Empires und Epochen, um den deutschen Fall besser verorten zu können (Abschn. 2). In den darauffolgenden Teilen wird die Produktion von Vergleichbarkeit in deutschen Kolonialstatistiken zunächst in Bezug auf das Territorium untersucht (Abschn. 3), später in Bezug auf Bevölkerungsgruppen (Abschn. 4). Der statistischen Beobachtung der Kolonien lag eine territoriale Grundunterscheidung zwischen Metropole und Kolonien zugrunde. Diese territoriale Unterscheidung war mit einer grundsätzlichen Unvergleichbarkeit zwischen kolonisierter Bevölkerung und Kolonisierenden eng gekoppelt, die in der Verwendung unterschiedlicher Methoden zur Erfassung dieser beiden Gruppen mündete.^13^ Der letzte Teil bündelt die Ergebnisse und skizziert Erklärungsansätze für den asymmetrischen Einsatz von Bevölkerungsstatistiken im deutschen kolonialen Kontext (Abschn. 5). Der Beitrag zielt somit auf eine empirisch-gestützte und historisch fundierte Reflexion des Vergleichs als soziale und wissenschaftliche Praxis sowie auf dessen Rolle in der Reproduktion gesellschaftlicher Ordnungsmodelle ab.

## Bevölkerungsbeobachtungen und Kolonialpolitik

Im 17. Jahrhundert wurden europäische Auswanderer auf der Reise in die britischen Kolonien Amerikas in Passagierlisten und bei der Ankunft registriert. In der britischen Kolonie Virginia findet man eine namentliche Liste der „Lebenden und Verstorbenen“ für das Jahr 1623 – sporadisch wurden auch versklavte Afrikaner und Indigene eingetragen, allerdings ohne Namen (Hotten 1874, S. 169 ff.; vgl. auch Wells 2015 [1975]). In der französischen Kolonie Neufrankreich in Nordamerika soll im Jahr 1666 eine Zählung stattgefunden haben, jedoch nur der französischen Siedler, nicht der indigenen Bevölkerung – ging es der französischen Regierung doch ausschließlich darum, die Entwicklung der Siedlergesellschaft zu verfolgen und zu unterstützen (Hacking 1982, S. 289). Im 18. Jahrhundert wurde die Bevölkerung (in vielen Fällen ausschließlich Siedler) in den kolonialen Empires regelmäßig ermittelt, meistens auf der Grundlage solcher Listen oder Register, punktuell auch mittels direkter Zählungen.^14^ Für das 19. Jahrhundert, als der Zensus zunehmend mit Exaktheit und Vollständigkeit assoziiert wurde und sich als bevorzugtes Instrument zur Wissensgenerierung über die Bevölkerung in Europa etablierte, lassen sich vereinzelte Versuche erkennen, sich dieses Instrument auch in den Kolonien zunutze zu machen. Ab den 1820er-Jahren und im Zuge der Unabhängigkeitsbewegung in Lateinamerika erklärte das spanische Kolonialreich „exakte Kenntnisse“ für notwendig, um die koloniale Herrschaft aufrechtzuerhalten (Aguilera 2017, S. 14). So wurden 1841 in Kuba eine zentrale und mehrere lokale Zensuskommissionen gebildet, die mit der Zählung der Bevölkerung (*censo de población*) beauftragt wurden und dabei Informationen aus erster und zweiter Hand kombinierten (Aguilera 2017, S. 20 ff.). Auch in Indien lässt sich nach dem Aufstand von 1857 eine ähnliche Entwicklung feststellen: Nachdem in den frühen 1850er-Jahren in einigen Regionen regelmäßig Zählungen unternommen worden waren (allerdings mit geringem Erfolg), wurde der erste All-India Census im Jahr 1871–1872 durchgeführt, der sich im 10-jährigen Rhythmus wiederholte und auf die systematische Erfassung aller Teile der indischen Bevölkerung abzielte (Leonhard und von Hirschhausen 2011, S. 70; Cohn 1987). Erst nach dem Aufstand war die Kolonialverwaltung nämlich zu dem Entschluss gekommen, man müsse die indische Bevölkerung besser kennen, um die koloniale Herrschaft auf Dauer zu sichern (Leonhard und von Hirschhausen 2011, S. 68 f.; Bayly 1996, S. 315–337).

In den meisten Kolonien jedoch blieb der All-India Census in seinem Anspruch, alle Teile der Bevölkerung im ganzen Land systematisch und nach den gleichen Kategorien zu erfassen, bis in die Zwischenkriegszeit (und darüber hinaus) ohne Äquivalent.^15^ James Duminy (2017) hat für Kenia gezeigt, wie sporadisch Zählungen der Bevölkerung zu Beginn des 20. Jahrhunderts stattfanden und wie begrenzt das statistische Interesse, abhängig vom jeweiligen politischen Kontext, war. Kolonisierte blieben meist von der statistischen Beobachtung ausgeschlossen – bis sie in der Zwischenkriegszeit im Zuge des sogenannten „Arbeitermangels“ in den Blickwinkel der kolonialen Regierung rückten (Duminy 2017).^16^

Auf den Treffen des Internationalen Statistischen Kongresses wurden zwar vereinzelte Fragen betreffend der „statistique coloniale“ (Quételet 1873, S. 90) diskutiert, jedoch keine systematischen Methoden oder Klassifikationen vereinbart. Grundsätzlich wurde statistisches Wissen über die Kolonien von den Teilnehmenden als wünschenswert erklärt. Allerdings war man sich der Grenzen eines solchen Wissens durchaus bewusst: So schlug ein Antragsteller in Den Haag (1869) vor, zwischen „Graden“ der Erkenntnisse zu differenzieren und das „Addieren von Zahlen unterschiedlicher Gewissheitsgrade zu vermeiden“ (Quételet 1873, S. 90, eigene Übersetzung). In einem weiteren Antrag wurden die Kolonialmächte aufgefordert, „eingeborenes Personal“ zum Zweck des Zensus auszubilden, „ausreichend“ zu entlohnen und „wertzuschätzen“ (Quételet 1873, S. 96). Es sei im Eigeninteresse der Kolonialmächte, diesbezüglich „großzügig“ zu sein.

In diesem imperialen und internationalen Kontext lassen sich die deutsche Kolonialpolitik und ihre Bevölkerungsbeobachtung verorten. Am 28. November 1885 kündigte Bismarck vor dem Reichstag den Beginn einer staatlich geförderten und geführten Kolonialpolitik an. Wenige Monate später folgten die ersten Denkschriften, die dem Reichstag zur Abstimmung über den Etat der Kolonialverwaltung – die einzige Kontrollmöglichkeit des Parlaments in Fragen der Kolonialpolitik – vorgelegt wurden.^17^

Ähnlich den Staatsbeschreibungen des späten 18. Jahrhunderts kombinierten die „Denkschriften über die Schutzgebiete“ verschiedene Medien und Wissensregister: Sie enthielten sowohl qualitative als auch quantitative Beobachtungen.^18^ Am Anfang der Untersuchungsperiode wurden in den Denkschriften oftmals Zahlen im Fließtext integriert. Erst nach 1900 nahmen Tabellen eine wichtigere Rolle ein und wurden seit 1902 aus den Beschreibungen extrahiert und in einem getrennten Anhang gesammelt.^19^

Im Folgenden werden diese Dokumente als historisches Artefakt der Kolonialverwaltung betrachtet (Stoler 2009) und nicht als Datenreservoir über die Kolonien, ihre Ökonomie oder ihre Bevölkerung analysiert. Am Beispiel der Produktion von Statistiken wird das besondere Verhältnis zwischen Souveränität, Territorium und Bevölkerung im kolonialen Kontext sichtbar.

## (Un‑)Vergleichbare Territorien

Das Territorium ist nicht bloß geografischer Raum, sondern auch ein politischer Raum, über den politische Autorität ausgeübt oder beansprucht wird (vgl. Schlögel 2006 [2003]). Folgt man historischen und soziologischen Analysen der Staatsbildung, so setzte moderne Staatlichkeit einen abstrakten Raumbegriff voraus, der sich in einer „functional equivalence“ der regierten Territorien ausdrückt (Joyce 2002, S. 98). Dieser Verstaatlichungsprozess ging mit einer Territorialisierung des Staatsgebiets einher; der Standardisierungsprozess, unter anderem die Benennung und Vermessung der als äquivalent angesehenen Gebiete, zielte auf eine zunehmende Integration der Territorien in den Staat ab (vgl. Brian 1994; Joyce 2002, S. 101). Der Zensus setzte eine Serie von territorialen Untereinheiten voraus, die als vergleichbar angesehen wurden. Zahlen, die auf der lokalen Ebene gewonnen wurden, mussten auf der nächsten administrativen Ebene aggregierbar sein – im deutschen Kontext zunächst auf bundesstaatlicher Ebene, um später Ergebnisse auf der Reichsebene zu liefern. Dieser Prozess stellte für die mit dem Zensus beauftragten Behörden und alle daran beteiligten Akteure eine besondere Herausforderung dar.^20^

Nun waren die meisten europäischen Staaten Ende des 19. Jahrhunderts nicht nur Nationalstaaten, sondern auch koloniale Empires. Wie gestaltete sich das Verhältnis zwischen Souveränität, Territorialität und Vergleichbarkeit im kolonialen Kontext? Mit welchen besonderen Herausforderungen waren die Akteure konfrontiert, um die Vergleichbarkeit diverser Gebiete herzustellen?

### Die imperiale Spaltung des Raums

Der Territorialisierungs- und Standardisierungsprozess ging mit dem Aufkommen neuer territorialer Kategorien und Differenzierungen einher. Im Laufe des Staatsbildungsprozesses verloren lokale Zugehörigkeiten zunehmend an Relevanz, während die nationale Ebene (oder die imperiale im deutschen Fall) an Bedeutung gewann. Es kam zu einer neuen territorialen Aufteilung zwischen Nationalgebiet, Ausland und Kolonien, die sich erst um 1900 stabilisierte.

Dies wird in den Debatten über den Rechtsstatus der deutschen Kolonien (offiziell „Schutzgebiete“^21^ genannt) sichtbar, die unter Juristen jahrzehntelang andauerten. Der unausgesprochene Kompromiss, der sich an der Praxis ablesen lässt, kann folgendermaßen zusammengefasst werden: Rechtlich gehörten die Gebiete der Kolonien zwar zum Reich und unterstanden der Hoheitsgewalt des Kaisers. Da sie aber außerhalb der physischen Grenzen des Reichs lagen, unterlagen sie besonderen, von Reichsgesetzen abweichenden Rechtsnormen.^22^ „Schutzgebiete“ wurden *de facto* als nicht ausländisches Gebiet mit separatem Rechtsraum definiert.^23^

Innerhalb des imperialen Raums fand folglich eine starke Differenzierung zwischen Metropole und Kolonien statt, die im Recht, aber auch im politischen und administrativen System und in statistischen Darstellungen verankert wurde. In der Verwaltung waren koloniale Angelegenheiten von inländischen Angelegenheiten strikt getrennt: In den ersten Jahren der deutschen Kolonialpolitik wurden sie dem Auswärtigen Amt in der Form einer „Kolonial-Abteilung“ (ab 1890)^24^ zugeordnet, bevor 1907 – im Zuge der „Kolonialreform“^25^ – das Reichskolonialamt gegründet wurde. In diesem Zusammenhang beschreibt George Steinmetz (2008) den Kolonialstaat als „semi-autonomous field“.

Aus dieser administrativen Trennung entstand auch eine besondere Arbeitsaufteilung in Bezug auf die Herstellung von Statistiken. Das Kaiserliche Statistische Amt (KSA, gegründet 1872) war exklusiv für die statistische Erhebung und Auswertung auf dem Reichsgebiet zuständig. Zahlen bezüglich der Kolonien wurden von den jeweiligen Gouvernements oder lokalen Verwaltungsebenen produziert und an die Reichsverwaltung in Berlin übermittelt. Diese administrative Differenzierung und die Trennung der Akteure bildete die Grundlage für den Einsatz von unterschiedlichen Zählverfahren.^26^ Außerdem wurde die Bevölkerung in der Metropole und in den Kolonien nach unterschiedlichen Prinzipien klassifiziert: Während der Zensus auf dem Gebiet des Deutschen Kaiserreichs seit 1890 zunehmend zwischen „Reichsangehörigen“ und „Reichsausländern“ (auf Basis der Staatsangehörigkeit) unterschied, galten in den Kolonien andere Kriterien zur Personendifferenzierung.

Metropole und Kolonien wurden somit als nichtäquivalente Räume betrachtet. Sie waren insofern inkommensurabel, als keine kategoriale und numerische Äquivalenz zwischen beiden Entitäten vorlag. In den statistischen Jahrbüchern beispielsweise sind Statistiken für die Metropole nach Sektoren in verschiedenen Kapiteln geordnet (Bevölkerung, Wirtschaftsstatistik, Bildung, Justiz etc.); das letzte Kapitel ist den Kolonien gewidmet, eine thematische Ausdifferenzierung erfolgte innerhalb des Kapitels.

Aber auch innerhalb der Kolonien fanden bestimmte Differenzierungen statt. Die Sozialordnung, die dem Kolonialprojekt innewohnte, drückte sich in einer besonderen Raumordnung aus (Jureit 2015, S. 11; vgl. auch Jureit 2016). Gegenstand der sogenannten „Schutzverträge“^27^ war unter anderem die Bestimmung des Gebiets, für das die „Hoheitsrechte“ von Europäern beansprucht wurden^28^ und das mithilfe landschaftlicher Markierungen (Fluss, Meer, Berggipfel, Stadt) definiert wurde (Coret 2019, S. 62). Eine zentrale Technologie zur Messung, Darstellung und Standardisierung des Raums stellten die Beobachtungen von Geografen dar, die in zahlreichen Expeditionen seit dem späten 19. Jahrhundert und systematisch nach der Gründung der „Kommission zur landeskundlichen Erforschung der deutschen Schutzgebiete“ 1905 die deutschen Kolonien erkundeten (Gräbel 2015). Dabei war die Zeit, die man zwischen zwei Punkten zu Fuß brauchte und die in Routenaufnahmen festgehalten wurde, ein wichtiger Maßstab, um den Raum zu erfassen und um Räume zum Zwecke der kartografischen Darstellung miteinander vergleichbar zu machen (Gräbel 2015, S. 197 f.). Die Uhr war ein genauso wichtiges Instrument wie der Kompass.

Die Herstellung von Vergleichbarkeit im kolonialen Kontext war streng mit den „Differenzwahrnehmungen“ verbunden, die für die Moderne und den Kolonialismus konstitutiv sind: „Ungleichheit war im kolonialen Diskurs eine Notwendigkeit, die man nur selektiv beseitigen zu müssen glaubte“ (Speich Chassé 2015, S. 588). Es herrschte eine „fundamentale Trennung zwischen Zentrum und Peripherie“, wobei bestimmte Territorien in der Peripherie aufgrund von Distanzen, Verkehrswegen und Personalmangel nicht durchgängig regierbar gemacht werden konnten (Speich Chassé 2015). Städte (in Afrika insbesondere Küstenstädte wie Dar es Salaam) waren „Inseln von Herrschaft“, die weitgehend vom Rest der Kolonie getrennt waren und anders regiert wurden (Pesek 2005). Die Kolonie Deutsch-Ostafrika beispielsweise war „weit davon entfernt, ein Territorium von Herrschaft zu sein“ (Pesek 2005, S. 18): „Jenseits der kolonialen Zentren blieb die Transformation des Raumes zu einem kolonialen Herrschaftsraum ein mehr als unvollendetes Projekt“ (Pesek 2005, S. 18–20).^29^

Die Stabilisierung des Territoriums war eine andauernde (und vergebliche) Beschäftigung der Kolonialverwaltung. Imperiale Projekte können keinesfalls als lineare Rationalisierung des Raums gedeutet werden – etwa von der „Entdeckung“, Eroberung bis zur Inanspruchnahme und unbegrenzten, vollständigen Kontrolle über koloniale Gebiete –, sondern waren bruchstückhafte, ungleichmäßige und unvollständige Unternehmungen, die auf der Gleichzeitigkeit von unterschiedlichen Rechtsnormen beruhten (Benton 2009).^30^ In Anlehnung an Jane Burbank und Frederick Cooper (2010) sowie Lauren Benton beschreibt Isabelle Surun (2019, S. 25) die kolonial-imperiale Herrschaft über Territorien und Bevölkerungen als ein Regime der „geschichteten (*feuilletée*)“ Souveränität, in dem die Autorität delegiert, geteilt, segmentiert wurde und oftmals zu Überlappungen führte.

### Von Mahenge^31^ nach Berlin: Zur Überbrückung von Distanz und Heterogenität im kolonialen Kontext

Nach 10 Jahren militärischer Machtausübung (1885–1895) wurde auch die deutsche zivile Kolonialverwaltung allmählich aufgebaut und in drei Ebenen gegliedert: die Zentralverwaltung in Berlin (Kolonial-Abteilung und später Reichskolonialamt), die Zentralregierung (Gouvernement) jeder Kolonie (unter der Leitung eines Gouverneurs) und die lokalen Verwaltungsstellen (ehemalige Militärstationen unter ziviler Herrschaft). Die Kolonien waren ihrerseits in Bezirke unterteilt, die Bezirke wiederum in Unterbezirke, Nebenstellen und (Militär‑)Stationen. Aus finanziellen Gründen blieb die personelle Besetzung aller Ebenen über den ganzen Zeitraum sehr gering (Eckert und Pesek 2004). In den lokalen Stellen folgte die administrative Aufgabenteilung keiner thematischen Spezifizierung.

Ab 1886 bestellte das für die Kolonialpolitik zuständige Dezernat in Berlin „Denkschriften über die Lage der deutschen Schutzgebiete“, die dem Reichstag als Vorlage zur Besprechung und Abstimmung des Etats vorgelegt werden sollten. Die an den Reichstag gesendete Denkschrift war eine Sammlung lokaler (qualitativer und quantitativer) Beobachtungen über Bevölkerung, politische Situation, wirtschaftliche Verhältnisse usw. Wie bei der Organisation des Zensus in der Metropole waren auch bei der Redaktion der Denkschrift alle Ebenen der (Kolonial‑)Verwaltung beteiligt. Die handschriftlichen Berichte der Gouverneure wurden dem Dezernat (später Kolonial-Abteilung oder Reichskolonialamt) übermittelt, das alle Informationen zentralisierte und dann den Schlussbericht für den Reichstag erstellte.

Bald entstand zwischen lokaler und zentraler Verwaltung eine Diskussion über die Frist für die Zusendung der Berichte und den Zeitraum der Berichterstattung. Zunächst einigte man sich auf das Kalenderjahr – ab dem Berichtsjahr 1900/1901 auf das Haushaltsjahr (1. April bis 31. März des Folgejahrs), wodurch der Verwaltung in den Kolonien drei zusätzliche Monate zur Fertigstellung der Berichte blieben. Hinter diesen Auseinandersetzungen über die *Temporalität* der Berichterstattung verbargen sich Fragen, die mehr mit kolonialer *Räumlichkeit* und Raumordnung zu tun hatten. Die Bitte nach der Gewährung einer zusätzlichen Frist seitens der lokalen Verwaltungsstellen lag in den wiederholten Verzögerungen in der Kommunikation zwischen den verschiedenen Verwaltungsebenen begründet. Im Empire mussten immense Distanzen unter beschränkten Möglichkeiten hinsichtlich Kommunikationswegen und Verkehrslage überbrückt werden (Steinmetz 2008, S. 592). An den Denkschriften lässt sich somit die bürokratische Anstrengung ablesen, sowohl auf die Zeit als auch auf den Raum einzuwirken.

Zu Beginn der Berichterstattung wurde die Übereinstimmung der Beobachtungen innerhalb einer Kolonie unter den unterschiedlichen Berichterstattern noch als Empfehlung von Berlin ausgesprochen: „Für die Kolonialverwaltung ist es von Interesse über den Zustand und die Entwicklung der einzelnen Schutzgebiete in übersichtlicher und *möglichst gleichförmiger* Weise regelmäßig unterrichtet zu werden“ (Kol.-Abt. (Kayser) 04.05.^1891^, eigene Hervor.). Aufgrund des dezentralen Produktionsprozesses und der vielen involvierten Akteure lief jede Ausgabe der Denkschrift Gefahr, sich als bloße Aneinanderreihung von disparaten Berichten und Sichtweisen ohne Bezug auf ein gemeinsames Drittes zu entpuppen. Zur Herstellung von Vergleichbarkeit (hier im Sinne von Uniformität) verfügte die Zentralverwaltung in Berlin über zwei Mittel: die Zusendung von Vorschriften für Klassifikation und Zählverfahren *vor* der Berichterstattung oder die Überarbeitung der einzelnen Berichte, *nachdem* sie in Berlin eingegangen waren. Am Anfang der Periode waren die Vorschriften aus Berlin eher spärlich – was zu aufwändigen Vereinheitlichungsarbeiten in den Gouvernements und in der Zentralverwaltung in Berlin führte. Ab 1900 war die Kolonial-Abteilung stets bemüht, einen einheitlichen Beobachtungsrahmen vorzugeben.

In einem Runderlass der Kolonial-Abteilung aus dem Jahr 1891 an die Gouvernements wurden lapidare Anweisungen in Bezug auf die erforderlichen Informationen und ihre Kategorisierung gegeben: „Bevölkerung. Anzahl der Europäer, unterschieden nach Nationalitäten und Berufsarten. Statistische Angaben über die Eingeborenen, soweit solche sich beschaffen lassen. Ein- und Auswanderung, soweit vorhanden“ (Kol.-Abt. (Kayser) 04.05.^1891^). Über die Art und Weise, wie statistische Angaben zu beschaffen waren, verlor die Kolonial-Abteilung kein Wort. Den lokalen Verwaltungsstellen wurde demnach viel Freiheit bei der Operationalisierung eingeräumt. Nur aggregierte Ergebnisse wurden in den Berichten an die nächsthöhere Ebene weitergereicht; wie die Daten tatsächlich generiert wurden (mithilfe von Zählkarten, Personenregistern etc.) und die Kalkulation erfolgte, wurde nicht mitgeteilt.^32^

Die Berichte der Bezirksämter folgten keiner formellen Struktur, die sich in Form von Titeln und Untertiteln ausdrücken würde. Es handelte sich um einen Fließtext mit wenigen Tabellen, die Bevölkerungszahlen gingen inmitten einer Lawine von Beschreibungen über spezifische und lokalisierte Ereignisse verloren. Der Bericht war in einer für jeden Produzenten spezifischen Sprache verfasst, ohne terminologischen Konventionen zur Beschreibung eines Ereignisses oder Phänomens zu folgen. Die Darstellung ähnelte dabei dem Reisebericht eines Administrators beim Besuch der verwalteten Gebiete. Im Bericht schrieb sich somit die Perspektive des Stationsleiters oder Bezirksamtsmanns ein, die weder systematisch noch abstrakt, sondern tief in lokalen Gegebenheiten verwurzelt und räumlich strukturiert war.

Sobald die Vorberichte der Bezirksämter im Gouvernement eintrafen, beschäftigte sich das zuständige Referat damit, diese für den Bericht des Gouverneurs auf der Ebene der Kolonie zu harmonisieren, um den Vorschriften aus Berlin zu entsprechen. Zu diesem Zweck wurden die Bezirke gebeten, zwei Exemplare des Berichts zu liefern. Eine Kopie wurde als solche aufbewahrt, das zweite Exemplar wurde zerschnitten und mit den Berichten der anderen Bezirke neu zusammengesetzt. Im Archiv des Reichskolonialamts ist die Materialität dieser Operation heute noch spürbar.^33^ In Berlin wurde dieser Vorgang wiederholt, nachdem alle Berichte der Gouverneure eingetroffen waren. Schließlich wurden die Einzelberichte in ein Gesamtdokument überführt und ab 1900 auch redaktionell überarbeitet – insbesondere um die Kategorien zu harmonisieren. Textstellen wurden entfernt oder geschwärzt. Durch diese Tätigkeiten verwandelte sich die Kolonial-Abteilung in eine bürokratische Maschinerie, die das Ziel verfolgte, einen Äquivalenzraum zwischen den Kolonien zu schaffen. Die finale Fassung der Denkschrift, die letztendlich dem Reichstag vorgelegt wurde, war das Ergebnis einer Vielzahl an Verfahren verschiedener Akteure, die an verschiedenen Orten die Vergleichbarkeit von heterogenem Material und lokalen Beobachtungen überhaupt erst herstellten und zugleich versuchten, diese auf Dauer zu sichern.

Im Zuge der Vereinheitlichung bürokratischen Handelns wurden ab 1900 erneut Anstrengungen unternommen, die Vergleichbarkeit *vor *der Datenerhebung zu gewährleisten. 1903 schickte die Kolonial-Abteilung den Gouverneuren Anweisungen („Grundsätze, betreffend die statistischen Erhebungen über die weiße Bevölkerung der Schutzgebiete in Afrika und der Südsee“)^34^ zur Terminologie und Klassifikation. Bereits der Titel des Schreibens macht deutlich, dass diese Regeln nicht für die Erfassung aller Bewohner einer Kolonie gelten sollten, sondern nur für die Gruppe der Siedler, die stets als „weiß“ kategorisiert wird. Somit wurden nicht alle Teile der Bevölkerung als gleich zählwürdig empfunden.^35^

Die als mangelhaft empfundene Bevölkerungsstatistik der Kolonie sollte eine „Neuordnung“ erfahren: „[E]s fehlte ihr vor allem die erforderliche Einheitlichkeit, welche sowohl zur Vergleichung der Bevölkerungsverhältnisse der einzelnen Schutzgebiete untereinander, als auch zur Vergleichung der Verhältnisse eines und desselben Schutzgebiets zu verschiedenen Zeitpunkten erforderlich ist“ (Kol.-Abt. des Auswärtigen Amts 1903, S. 409). Die Homogenität, Kontinuität und Vergleichbarkeit der Kolonialstatistiken sollte durch die Standardisierung (1) des zeitlichen Vergleichs- und Beobachtungsrahmens, (2) der Beobachtungseinheit (Definition der zu erfassenden Personen) und (3) der Beobachtungskriterien (Nomenklaturen) für die gesamten Kolonien erreicht werden. Die „weiße“ Bevölkerung sollte stets zum 1. Januar festgestellt werden und es sollten nur jene Personen, die in den Kolonien „ansässig“ (und nicht „zufällig anwesende“ Personen) waren, eingeschlossen werden. Die Nomenklaturen wurden von Berlin aus festgelegt und in Form von Mustertabellen in die Kolonien verschickt. Die Zusendung genauerer Vorschriften wurde von der Kolonial-Abteilung als ein Weg zur Qualitätsverbesserung von Statistiken betrachtet, ohne jedoch in die statistische Ausbildung des Personals zu investieren oder die personelle Besetzung aufzustocken und damit weitere Kosten zu generieren.

Nach der Gründung des Reichskolonialamts im Jahr 1907 wurde ein weiterer Versuch unternommen, die Heterogenität der Berichte zu reduzieren. In einem Entwurf eines Runderlasses wurde der Wert quantitativer Beobachtungen zur Erhöhung der Vergleichbarkeit besonders hervorgehoben. Indem sie qualitative Ereignisse in eine gemeinsame Sprache übersetzen und die Zahl als Konvention nehmen, schienen Statistiken besonders gut geeignet zu sein, den Denkschriften einen einheitlichen Charakter zu geben: „Alle die Ausführungen, welche Tatsachen aus der Berichtszeit vorbringen, … namentlich wenn sie durch die ziffernmässigen Tatsachen belegt sind, machen die Berichterstattung besonders wertvoll. … Wo immer es zweckentsprechend ist, muss die Darstellung basiert sein auf einem Vergleich der neuen Berichtstatsachen mit den analogen Verhältnissen der früheren Jahre oder wenigstens des Vorjahres, da man in vielen Fällen nur auf diese Weise zu einer richtigen Beurteilung und zu brauchbaren Schlüssen gelangen kann“ (Reichskolonialamt 03.07.^1907^). Statistiken sollten in den Berichten nicht nur zur Beschreibung einzelner Situationen in den Kolonien verwendet werden, sondern auch zur Beschreibung von dynamischen Entwicklungen aufgrund von zeitlichen Vergleichen und schließlich auch um Ursache-Folge-Beziehungen zu beleuchten. Dieser Entwurf, vom Mitarbeiter des Wirtschaftlichen Referats und promovierten Nationalökonomen Gottfried Zöpfl bearbeitet, wurde vom Direktor des Reichskolonialamts Bernhard Dernburg als zu anspruchsvoll eingeschätzt und schließlich nicht abgeschickt.

In Berlin versuchte die Kolonialverwaltung gleichzeitig Äquivalenz *zwischen* den Kolonien und *innerhalb* der Kolonien zu schaffen. Bis 1900 waren die quantitativen und qualitativen Beobachtungen in den Berichten kaum vorstrukturiert und die Heterogenität des Endprodukts wurde bemängelt. Angeregt durch die Kolonial-Abteilung in Berlin und die Gouverneure setzte nach 1900 eine Konventionalisierung in Bezug auf Territorien und Bevölkerungsgruppen ein. Wie sich diese Vereinheitlichungsversuche auf der Ebene der Kategorien ausdrückten, ist Gegenstand des nächsten Teils.

## (Un‑)Vergleichbare Bevölkerungsgruppen

Während in der Metropole (s. Heintz 2021; Wobbe 2021) innerhalb der Gesamtbevölkerung Ähnlichkeiten und Differenzen entlang von bestimmten Kriterien konstatiert wurden, wurde in den Kolonien eine grundlegende Andersartigkeit zwischen Kolonisierten und Kolonisierenden postuliert und wurden unterschiedliche Zählverfahren für beide Gruppen veranlasst. „Europäer“ und „Eingeborene“ – so die offiziellen Kategorien – wurden als grundsätzlich nichtäquivalent und somit als unvergleichbar angesehen. Somit fehlte die Voraussetzung für die Herstellung eines Vergleichsrahmens, nämlich die Annahme einer grundsätzlichen Gleichartigkeit der Einzelnen als Mess- und Untersuchungseinheit. Durch die Verwendung unterschiedlicher Methoden für die Kolonisierenden einerseits und die kolonisierte Bevölkerung andererseits wurde diese Unvergleichbarkeitsannahme nicht nur reproduziert, sondern überhaupt erst begründet, in Zahlen verfestigt und in bildlichen Darstellungen (den Tabellen) verankert, so mein Argument. Dieser Prozess wird im Folgenden als *methodologische Alterität *bezeichnet.

### Methodologische Alterität

Die Vorschriften der Zentralverwaltung in Berlin enthielten klare Anweisungen in Bezug auf die zu erhebenden Merkmale der „europäischen“ Bevölkerung – zumindest nach 1900. Dies war für die einheimische Bevölkerung nicht der Fall, wenngleich ab 1891 statistische Angaben erwünscht waren. Für beide Gruppen waren jedoch die Verordnungen aus Berlin hinsichtlich der Zählmethoden äußerst unpräzis; wichtig war nur das Ergebnis, nämlich dass die Denkschriften in irgendeiner Form auf quantitativem Wissen basierten.

Aus den gesichteten Quellen wird nicht klar, wie die „europäische“ Bevölkerung gezählt wurde: ob durch grobe numerische Schätzungen oder auf der Grundlage von Registern.^36^ Es ist durchaus denkbar, dass in manchen Fällen Registerdaten verwendet wurden und in anderen Fällen Zählungen durchgeführt wurden.^37^ Die Erhebungspraktiken wichen nicht nur von Kolonie zu Kolonie voneinander ab, sondern auch innerhalb einer Kolonie von Bezirk zu Bezirk (und teilweise auch von Jahr zu Jahr).

Zahlen über die kolonisierte Bevölkerung wiederum blieben in den ersten Denkschriften gänzlich aus. In einer nächsten Phase folgten Schätzungen, allerdings nicht für das gesamte Territorium einer Kolonie, sondern meist nur bezogen auf Städte oder Küstenbezirke. Diese Schätzungen gründeten auf einem Koeffizienten (der aus einer durchschnittlichen Einwohnerzahl pro Hütte oder aus der steuerpflichtigen Bevölkerung abgeleitet wurde, wie beispielsweise in Deutsch-Ostafrika für das Berichtsjahr 1912/1913). In einigen Fällen wurden Zählungen auch mittels Steinen (1 Stein = 1 Einwohner, wie auf den Marschallinseln im Jahr 1908) oder Getreide (wie in Togo) durchgeführt. Es handelte sich also überwiegend nicht um einen *individuellen *Zensus, der auf die Erhebung personenbezogener Merkmale abzielte, sondern um rein *numerische* Aufzählungen, welche die Feststellung der Gesamtzahl der Bevölkerung eines Territoriums intendierten (Gervais und Mandé 2007, S. 65).^38^ Die Auswahl der Methode macht sichtbar, dass die kolonisierte Bevölkerung eher als undefinierte Masse denn als Summe einzelner Individuen gesehen wurde.^39^

Den extrem detaillierten Beschreibungen der Kolonisierenden stand die Approximation als Methode zur Beobachtung der Kolonisierten gegenüber. Dieses Missverhältnis liegt in den ungleichmäßigen Maßnahmen zur Messung der beiden Gruppen begründet. Durch die Ungleichmäßigkeit der Beobachtung wurde die Gruppe der Kolonisierenden unverhältnismäßig präzise dargestellt und sichtbar, während die restlichen Einwohner, die die überwiegende Mehrheit darstellten, nicht erfasst und somit invisibilisiert wurden (von Trotha 1994, S. 394).

Die Ergebnisse dieser unterschiedlichen Zählverfahren wurden in den Denkschriften in separaten Tabellen für die „europäische“ Bevölkerung und die „eingeborene“ Bevölkerung festgehalten. Im Unterschied zu Statistiken zu den französischen oder britischen Kolonien findet sich keine Gesamtsumme der unterschiedlichen Gruppen. Das hatte zur Folge, dass keine statistische Vorstellung über die Gesamtbevölkerung der Kolonien entworfen wurde. Dafür hätten die Messeinheiten – die Individuen – zumindest partiell als gleich betrachtet werden müssen. Hierin besteht ein grundlegender Unterschied zu den statistischen Verfahren und Wissensgenerierung über die Bevölkerung in der Metropole.^40^

### Koloniale Klassifikationsprinzipien

Bis jetzt wurde von einer grundsätzlichen Unterscheidung zwischen Kolonisierenden und Kolonisierten in den Statistiken ausgegangen, die sich in getrennten Zählverfahren und Tabellen ausdrückte. Aber welche Kategorien wurden von der Kolonialverwaltung benutzt, um die Bevölkerung zu gliedern? Wie stabil waren die Nomenklaturen über den untersuchten Zeitraum? Während zu Beginn der Periode eine Vielzahl an Personenkategorien zur Verfügung stand, die von Bezirk zu Bezirk, von Kolonie zu Kolonie stark variierten, reduzierte sich der Wissenshorizont in allen deutschen Kolonien ab 1900 auf nur zwei Klassifikationsmöglichkeiten, „weiße“ vs. „farbige“ Bevölkerung.

Je nach Gebiet, Jahr oder Dokument schwankten vor 1900 sowohl die Anzahl der verfügbaren Kategorien als auch ihre Bezeichnungen. Anders als es das Kaiserliche Statistische Amt inzwischen für die Bevölkerungsstatistik in der Metropole praktizierte, schrieb die Zentralverwaltung in Berlin keine gemeinsame Klassifikation und Terminologie vor. In den ersten Denkschriften wurden Gruppen ohne systematische Klassifikation aufgezählt. Die Reihenfolge und Bezeichnungen änderten sich von Stadt zu Stadt und von Bezirk zu Bezirk. Für die Stadt Bagamoyo in Deutsch-Ostafrika wurden beispielsweise folgende Gruppen aufgelistet (und nicht in tabellarischer Form dargestellt): „Die Einwohnerschaft … setzt sich zusammen aus der Europäerkolonie (47 Personen), der indischen Kodjagemeinde (608 Personen) …, den Mastatarabern (70 Personen) …, der Polizeitruppe (35 Personen), dem Anhang der Soldaten (120 Personen) …“ (Denkschrift 1893, S. 390). Die Kategorisierung folgte nicht einem einzelnen Kriterium, sondern einer Mischung aus ethnischen und beruflichen Zuschreibungen.

Die Klassifikationsschemata waren einerseits durch rassistische Deutungsmuster geprägt, andererseits durch eine territoriale Logik. So wurden als „Fremde“ diejenigen Personen kategorisiert, die im Gegensatz zu „Eingeborenen“ als *nicht von dieser Kolonie *eingestuft wurden (ob sich diese Einschätzung auf den Wohnort, den Geburtsort oder ein anderes Kriterium stützte, wird in den Dokumenten nicht näher erläutert). Dies traf auf europäische Siedler zu, aber auch auf Personen, die aus einer Kolonie außerhalb des deutschen Kolonialreichs (z. B. Indien) sowie aus anderen unabhängigen Staaten (z. B. China) kamen. Darüber hinaus waren die Grenzen der „europäischen“ Bevölkerung in dieser ersten Periode höchst instabil. Mal waren „Goanesen“, mal „Türken“, mal „Syrer“, „Armenier“ oder „Amerikaner“ der Kategorie „Europäer“ zugeordnet, mal nicht (Hermann 1900/1901, S. 269). Außerdem sind in den Dokumenten die Labels „europäisch“ und „weiß“ bis zu Beginn des 20. Jahrhunderts austauschbar.

Der Standardisierungsprozess, der von der Kolonial-Abteilung in Berlin ab 1900 forciert wurde, äußerte sich auch in der Vereinheitlichung der Beobachtungsschemata. Der Terminus „weiße Bevölkerung“ wurde in einem Schreiben aus dem Jahr 1901 an die Gouverneure durchgängig verwendet, was wiederum Effekte auf die nächste Denkschrift hatte (Kol.-Abt. 1901). Für das Jahr 1901 wurde nach diesem Schema zum ersten Mal die Gesamtzahl der „weißen Bevölkerung“ in den deutschen Kolonien ermittelt (ca. 6500 „Köpfe“) (Denkschrift über das Berichtsjahr 1900/1901 (1902), S. 3125). Die Kategorie selbst ging von einer Äquivalenz der einzelnen Individuen aus. Sie fokussierte das Gemeinsame, während Unterschiede innerhalb der Kategorie (z. B. in Bezug auf ihre Staatsangehörigkeit) ausgeblendet wurden. Von diesem Zeitpunkt an wurden in den statistischen Anlagen zu den Denkschriften auch zusammenfassende Tabellen der Bevölkerung aller deutschen Kolonien veröffentlicht – die kolonisierten Bevölkerungsgruppen ausgenommen. Es wurden also alle Kolonien zusammengefasst, auf Zahlen zurückgeführt und dadurch vergleichbar gemacht, doch bestimmte Gruppen blieben aus dem Vergleich ausgeschlossen.

Dabei war es nicht eindeutig, welches Kriterium die Kategorie „weiß“ markieren sollte. In einem Runderlass der Kolonial-Abteilung aus dem Jahr 1902 wurde zwischen Nationalität, Kultur, geografischer Herkunft und Staatsangehörigkeit geschwankt – für jedes Kriterium wurden Vorteile und Nachteile aufgeführt. Schließlich hieß es: „Da unter diesem Begriff jedoch die *Gesamtheit der Träger unserer Kultur* in den Schutzgebieten erfaßt werden soll, bleibt nur übrig, daß man neben den in Europa ansässigen Völkern die in fremden Erdteilen geborene weiße Bevölkerung einbezieht, so die Amerikaner, Australier, Kapländer^41^ usw. Als Bezeichnung für den so abgegrenzten Begriff ist ‚weiße Bevölkerung‘ anzunehmen“ (Kol.-Abt. 08.01.^1902^, eigene Hervor.). Das Zitat verdeutlicht die Kategorisierungsarbeit und expliziert zugleich die getroffenen Entscheidungen, die hinter der Einführung der neuen Kategorie standen. Die Grenzen der Kategorie sind die der vorgestellten Kultur. Die Kategorie „weiß“ wird dafür als *Konvention* „angenommen“. Dies zeigt auch, dass im kolonialen Kontext zuerst Gruppen definiert wurden, *bevor* sich die Kolonialverwaltung über mögliche Klassifikationskriterien Gedanken machte.

Personenkategorien, die nicht in dieses Schema passten und die bisher an der Schnittstelle zwischen den Kategorien „Europäer“ und „Eingeborene“ eingestuft worden waren, mussten neu zugeordnet werden. Dabei erfuhr die Kategorie „Eingeborene“ ebenfalls eine Änderung: Sie wurde erweitert und in „Farbige“ umbenannt, um Angehörige anderer Empires (zuvor: „nichteuropäische Fremde“) sowie Nachkommen aus Ehen zwischen Kolonisierenden und Kolonisierten (als „Mischlinge“ genannt) hinsichtlich einer als relevant betrachteten Ähnlichkeit einzuschließen. Nur einige Jahre zuvor überwogen in den Augen der Kolonialbeamten noch die Unterschiede, sodass diese Gruppen unterschiedlichen Kategorien zugeordnet wurden.^42^ Die neue Definition und Konvention, die sich ab 1902 mit der Kategorie „weiße Bevölkerung“ durchsetzte, sowie die wachsende Relevanz „rassischer“ Deutungsmuster^43^ erforderten eine Revision des gesamten Klassifikationssystems. Auf der lokalen Ebene erweist sich dieser Prozess jedoch als brüchig und nicht eindeutig. Die Gouverneure sahen sich gezwungen, durch Rundschreiben an die Direktoren der lokalen Stellen auf das neue Klassifikationsschema und die daraus entstehenden Kategorien und Definitionen immer wieder hinzuweisen. Abweichungen zur vorgegebenen Klassifikation wurden in der finalen Fassung der Denkschrift durch die Kolonial-Abteilung und später das Reichskolonialamt in Berlin geglättet.

## Selektives Wissen und Asymmetrien

Die Ergebnisse zeigen die doppelte Unvergleichbarkeit der Bevölkerungsstatistiken im deutschen kolonialen Kontext: Auf der einen Seite wurden Metropole und Kolonien als unvergleichbar betrachtet, auf der anderen Seite wurden Kolonisierte und Kolonisierende als so grundlegend anders angesehen, dass unterschiedliche Methoden zur Zählung und Kategorisierung legitim erschienen.

Offen bleibt, warum Zahlen über Kolonisierte ein so seltenes Gut waren.^44^ Im Folgenden werden einige der Erklärungsversuche skizziert, die von zeitgenössischen Akteuren selbst angeführt wurden. Kolonisierte zu zählen, wäre mit einer Aufstockung an Personal, Strukturen und Technologien verbunden gewesen, die für die Kolonialverwaltung nicht infrage kam. Somit verfügten die Gouvernements in den Kolonien nicht über das notwendige statistische Wissen und die Techniken. Im Stab der Gouvernements befanden sich z. B. keine ausgebildeten Statistiker. Entgegen den Empfehlungen des Internationalen Statistischen Kongresses in Den Haag ist für die deutschen Kolonien unklar, ob – und wenn ja, wie – lokale Zähler rekrutiert wurden. Es herrschte seitens der Kolonialverwaltung eine generelle Skepsis gegenüber der lokalen Bevölkerung und den lokalen Autoritäten, denen man vorwarf, „unzuverlässige“ Angaben zu machen, die nicht kontrolliert werden konnten. Eine in den Quellen oft verwendete Argumentationslinie betrifft die den Kolonisierten zugeschriebene Unfähigkeit, mit Zahlen umzugehen.^45^ In diesem Diskurs wurden Zahlen mit „Zivilisation“ gleichgesetzt.^46^ Dazu kam, dass bestimmte Gruppen als nicht zählwürdig eingestuft wurden: Aus der Datenlage ließe sich kein ökonomischer Profit ziehen, da diese Gruppen sich laut der Kolonialverwaltung nicht als Arbeitskräfte „eignen“ würden.^47^ Schon lange vor Lucien Lévy-Bruhls *Primitive Mentalität* (1931)^48^ wurde den Kolonisierten von der deutschen Kolonialverwaltung Rationalität und Zählvermögen abgesprochen, was dazu führte, dass sie sowohl selten gezählt wie auch als potenzielle Zähler abgelehnt wurden. Zusammenfassend kann man festhalten, dass die desolate Lage betreffend Statistiken in den deutschen Kolonien von der Kolonialverwaltung sowohl durch die materiellen Mängel der Verwaltung selbst als auch durch die Charakteristika, die den Kolonisierten aufgrund rassistischer Stereotypisierung zugeschrieben wurden, begründet wurde.

Die Verwissenschaftlichung des Kolonialismus, die bereits im späten 19. Jahrhundert einsetzte, erfolgte „unvollständig, zögerlich und widersprüchlich“, wie Daniel Speich Chassé für das Britische Empire festhält (2015, S. 596). Vor allem aber waren nicht alle Natur‑, Sozial- und Geisteswissenschaften im gleichen Maße und in allen Phasen der Kolonisierung daran beteiligt.^49^ Um 1900 dominierte das ethnografische Wissen, das zum Teil von der Kolonialverwaltung selbst produziert wurde (Steinmetz 2007). In Bezug auf demografische Daten über die Kolonisierten stellen Denis D. Cordell et al. (2010, S. 7) Folgendes fest: „In the early years of European colonization, the violence and upheaval of conquest as well as the lack of administrators and resources overshadowed the collection and analysis of demographic data about newly vanquished populations. … Only in the 1920s and 1930s did European colonial authorities attempt to refine the enumeration of their subjects and apply demographic methods by then common in the metropole to the analysis of colonial populations“. Ab den 1930er- und 1940er-Jahren begann eine neue Phase der Kolonisierung, die von George Steinmetz als „colonial developmentalism“ beschrieben wird und die zunehmend den Beitrag der Ingenieurs- und Sozialwissenschaften zu nutzen suchte: In den britischen und französischen Empires wurden in der Folge nicht nur die Verwaltungsapparate in den Kolonien massiv ausgebaut und statistische Abteilungen geschaffen, sondern die „koloniale Situation“^50^ wurde auch zum Gegenstand anderer Disziplinen wie der Soziologie (Steinmetz 2017; Cooper 2004).

Vor dem Hintergrund meiner Ergebnisse ist es fraglich, inwieweit die deutsche Kolonialpolitik vor 1914 die *Regierung* der kolonisierten Bevölkerung (im Sinne von Foucaults „Biopolitik“) in Form einer Planung ihrer dynamischen Entwicklung mittels umfassender Zählungen zum Ziel hatte. Ziele waren vielmehr die militärische Kontrolle, die Ansiedlung deutscher Kolonialisten sowie die Ausbeutung der Rohstoffe und der Kolonisierten für Arbeitszwecke. Dafür konnte sich die Verwaltung auf andere Wissenschaften und Techniken, wie geografische Expeditionen, kartografische Erfassungen oder die „Tropenmedizin“, stützen. In den meisten Fällen beruhte die Kolonialherrschaft zusätzlich oder ausschließlich auf repressiven Maßnahmen, auf direktem, körperlichen Zwang und Gewalt.^51^ Hier zeichnet sich eine Herrschaftsform ab, die auf der „Elision“ des kolonialen Subjekts – der strategischen Ausblendung ganzer Gruppen beruhte (Luste Boulbina 2006, S. 197).^52^

In den deutschen Kolonien scheint das Interesse für die nichteuropäischen Bewohner der kolonisierten Gebiete seitens der Kolonialverwaltung sehr partiell und immer abhängig vom jeweiligen Kontext gewesen zu sein. Interessenswürdig wurden sie erst unter den folgenden Bedingungen: bei Widerstand, als potenzielle Arbeitskräfte auf den Plantagen (die aber noch zu solchen „erzogen“ werden müssten, vgl. Conrad 2004) und nach der Einführung der Kopfsteuer seit 1900 auch zunehmend als finanzielle Ressource (ab 1907 bzw. 1908 in Togo und Kamerun). Dies sollte sich erst ab 1907 mit der sogenannten „Kolonialreform“ unter Bernhard Dernburg ändern, die der sogenannten „Eingeborenenpolitik“ und dem Wissen über die Kolonisierten größeres Gewicht einräumte (vgl. Utermark 2012).^53^ Jedoch lassen sich auf der Ebene der Bevölkerungsstatistik bis zum Ende der deutschen Kolonialherrschaft keine bedeutenden Änderungen feststellen.

Auch wenn die Zählungen weit davon entfernt waren, vollständig und regelmäßig stattzufinden, lässt sich dennoch ein „unintendierter Effekt“ (Hacking 1982, S. 280) der statistischen Praxis festhalten. In der Suche der Kolonialverwaltung nach einer gemeinsamen Sprache und nach Äquivalenzen innerhalb des Empires entstanden neue Kategorien, die sich in der internen Kommunikation und in den Denkschriften, die der Öffentlichkeit vorgelegt wurden, zu neuen Konventionen entwickelten. Die Kategorien „weiße“ und „farbige“ Bevölkerung sowie ihre binäre Gegenüberstellung sind das Resultat dieses Prozesses. Sie werden noch weit über den Ersten Weltkrieg hinaus administrative Denkmuster und den öffentlichen Diskurs prägen.

## Fazit: Vergleichsverbote und Vergleichsgebote

Der Zensus als administrative Praxis sowie die vielen Operationen des Vergleichs, die ihm zugrunde liegen, lassen sich im Spiegel der Kolonialstatistik betrachten. Warum wurden Kolonisierte und Kolonisierende, Metropole und Kolonien im deutschen Kontext nicht miteinander verglichen? Die Gründe hierfür sind sowohl methodologischer und politischer als auch kognitiver Art.

In den deutschen Kolonien wurden Zahlen zwar als gemeinsame Sprache des Empires genutzt, um geografische Entfernungen und kulturelle Heterogenität zu überbrücken, doch die Vergleichbarkeit von räumlich diversen und entfernten Gebieten wurde nie dauerhaft hergestellt. Statt abstraktes Wissen hervorzubringen, zeugten die kolonialen Denkschriften in vielen Fällen von der Subjektivität der Berichterstatter und von der Lokalität der Beobachtungsperspektive. Ab 1900 versuchte die Kolonial-Abteilung, die Methoden und Klassifikationsschemata innerhalb des Empires zu vereinheitlichen, jedoch ohne die materiellen Bedingungen für eine Standardisierung zu schaffen, insbesondere ohne Personal einzustellen und auszubilden. Finanzielle Knappheit prägte die Kolonialpolitik und die Kolonialverwaltung durchgängig. Unter diesen Bedingungen begnügte man sich in Berlin mit Zahlen, deren Verlässlichkeit methodologisch nicht überprüfbar war. Man wollte die lokalen Verwaltungsstellen nicht mit nationalökonomischen Theorien überfordern. Die Qualität der Zahlen war zweitrangig, ihre Existenz hingegen, als Symbol staatlichen Handelns und Legitimation, war für die Kolonial-Abteilung in Berlin von zentraler Bedeutung.

Neben politischen und methodologischen Vergleichshürden gab es aber auch kognitive Hindernisse. Die räumliche Unvergleichbarkeit zwischen Metropole und Kolonien wie auch innerhalb der Kolonie war an ein Vergleichsverbot zwischen Kolonisierenden und Kolonisierten gekoppelt. Die Verwendung verschiedener Methoden für beide Bevölkerungsgruppen machte Vergleiche unwahrscheinlich; zugleich stellten diese Methoden statistische Inkommensurabilität erst her. Die Analyse kolonialer Praktiken wirft somit Licht auf das Vergleichs*gebot*, das dem Zensus innewohnt. Individuen als vergleichbare Untersuchungseinheiten anzuerkennen, ist das Ergebnis eines langen, gesellschaftlichen Prozesses, der Mitte des 18. Jahrhunderts mit der Liberalisierung westlicher Gesellschaften einsetzte: „Implicitly, at least, statistics tended to equalize subjects. It makes no sense to count people if their common personhood is not seen as somehow more significant than their differences“ (Porter 1986, S. 25). Gleichzeitig wurden ganze Teile der Welt und ihre Bewohner von diesem Prozess ausgeschlossen und mit einem Vergleichs*verbot* versehen. Die Ergebnisse zeigen, dass dieses Vergleichsverbot nicht nur auf rassistischen Deutungsmustern beruhte, sondern auch auf einer strukturellen, systematischen Trennung zwischen innenstaatlichen Regierungspraktiken und kolonialen Angelegenheiten, die einem anderen Rechtsregime unterworfen waren – wobei beide Arten von Differenzierung (rassistisch und territorial-rechtlich) eng miteinander verwoben waren und sich wechselseitig bedingten. Somit erlaubt die soziologische Analyse kolonialer Phänomene, Widersprüche und Grauzonen der Modernisierungs- und Globalisierungsprozesse auf neue Weise zu betrachten und dabei den Einsatz von Regierungstechnologien differenziert zu bewerten.

## Footnotes

1 In ihrer vergleichenden Studie fassen Burbank und Cooper unter „Politik der Differenz“ die unterschiedlichen Kombinationen von Einschluss- und Ausschlussmechanismen zusammen, die in imperialen Konstellationen zur Herstellung von (Un‑)Gleichheit beitragen (Burbank und Cooper 2010, S. 458).

2 Vergleiche jedoch Leonhard und von Hirschhausen (2011, S. 53 ff.), Ittmann et al. (2010) und Touchelay (2019).

3 „Kolonialstatistiken“ (im Plural) ist keine emische Kategorie, sondern wird hier verwendet, um Beobachtungen zu beschreiben, die von der Kolonialverwaltung über die (deutschen) Kolonien produziert wurden.

4 Zum Begriff Biopolitik und seiner Aktualität vergleiche Pieper et al. (2011); zum demografischen Projekt im kolonialen Afrika vergleiche Ittmann et al. (2010).

5 Dass im Folgenden das generische Maskulinum verwendet wird, ist die Entscheidung der Redaktion der KZfSS. Die Autorin hat den Text ursprünglich in einer inklusiven und geschlechtergerechten Sprache abgefasst.

6 Foucault selbst verliert in seinem Werk allerdings nur wenige Worte über den Kolonialismus. Über die Relevanz der Foucault’schen Theorie für die Analyse kolonialer und postkolonialer Kontexte vergleiche Stoler (1995), Scott (1995), Kalpagam (2014), Legg (2007), Gammerl (2010) und Isin und Ruppert (2019).

7 Im Folgenden ist vom Zensus „im Sinne einer quantitativen, auf Zahlen basierenden Dokumentation und Analyse der Zusammenhänge sozialer und wirtschaftlicher Verhältnisse des Staates“ die Rede (von Oertzen 2017, S. 413).

8 Diese sind als Anlagen zu den Reichstagsprotokollen auf https://www.reichstagsprotokolle.de/ abrufbar.

9 Heute Tansania, Burundi und Ruanda.

10 Heute Namibia.

11 Heute Papua-Neuguinea, Mikronesien, Nördliche Marianen, Palau, Nauru, Marshallinseln und Salomonen.

12 Die chinesische Kolonie Kiautschou, die vom Reichsmarineamt verwaltet wurde, war Gegenstand getrennter Berichte, die nicht in den analysierten Korpus aufgenommen wurden.

13 Im Folgenden werden die pauschalisierenden Begriffe „Kolonisierende“ und „kolonisierte Bevölkerungsgruppen“ oder „Kolonisierte“ als Sprachkonvention verwendet, um die Kontingenz und die Konstruktion binärer Kategorien im kolonialen Kontext zu betonen.

14 Zur Reform des administrativen Wissens in Angola unter der portugiesischen Herrschaft im 18. Jahrhundert vgl. Santos (2010).

15 An dieser Stelle sei auf die Veröffentlichung eines *Census of the British Empire, 1901* im Jahr 1905 hingewiesen (Great Britain, Census Office 1906). In dieser einmaligen Publikation versuchten britische Statistiker, basierend auf quantitativen Beobachtungen ein umfassendes Bild des Empires darzulegen. In vielen Fällen mussten sie sich jedoch mit groben Schätzungen oder gar Lücken begnügen, insbesondere in Bezug auf die kolonisierte Bevölkerung.

16 Im französischen Empire wurden ab 1856 in Algerien und 1891 in Tunesien regelmäßige Volkszählungen im gleichen Abstand und nach ähnlichen Prinzipien wie in der Metropole durchgeführt (Kateb 2004). Während sich die Erhebung in Algerien in einigen Regionen auch auf Teile der kolonisierten Bevölkerung erstreckte, wurde diese in Tunesien wiederum bis 1911 komplett ausgelassen (Renard 2021).

17 Die Berichterstattung für den Reichstag wurde 1909 unter anderem aus finanziellen Gründen ausgesetzt. In den Folgejahren erschienen noch „Jahresberichte“ als Veröffentlichung des Reichskolonialamts, das fortan für die Publikations- und Vervielfältigungskosten aufkommen musste. Im Fall kolonialer Statistiken oder Berichterstattung mussten Ausgaben gegenüber dem Gesetzgeber in der Metropole in doppelter Hinsicht gerechtfertigt werden: einerseits, weil hohe Kosten für statistische Operationen generell, auch in der Metropole und bis heute für Debatten im Parlament sorgen können; andererseits, weil die Abgeordneten sich in vielen Fällen für eine Senkung der staatlichen Finanzierung der Kolonialpolitik aussprachen – nicht nur im deutschen Kontext, sondern auch in anderen europäischen Empires.

18 Statistik mit Quantifizierung gleichzusetzen ist eine recht junge Entwicklung. Von der frühen Neuzeit bis in das 19. Jahrhundert hinein wurde Statistik im Sinne einer „Staatswissenschaft“ – gerade im deutschsprachigen Raum in der Tradition Gottfried Achenwalls – als eine Beschreibung des Staats verstanden, die unterschiedliche Formen annehmen konnte (Porter 1986, S. 24). Erst ab Mitte des 19. Jahrhunderts setzte sich ein rein numerisches Verständnis von Statistik und Tabellen als bevorzugte Darstellungsweise durch. Zur Geschichte der Statistik und zum Verhältnis zwischen beschreibender und numerischer Tradition in der frühen Neuzeit vergleiche auch Behrisch (2006, 2016); Berg et al. (2015) und Rassem (1980).

19 Die letzten Ausgaben der Denkschriften waren, inkl. statistischem Anhang, etwa 700 Seiten stark, während die erste Denkschrift aus dem Jahr 1885 nur vier Seiten lang war.

20 Alain Desrosières (1997, S. 55) erinnert an den ersten Zensus in Frankreich im Jahr 1800, der das jüngst eingeführte Verwaltungsnetz herausforderte und anhand von Berichten der lokalen Autoritäten (Präfekt) durchgeführt wurde. Die Berichte, die in Paris eintrafen, waren „wortwörtlich inkommensurabel, da die Perspektive jedes Präfekten so unterschiedlich gewesen war. Man wusste nicht, wie sie aggregiert werden sollten“ (Desrosières 1997, eigene Übersetzung).

21 Die Ambivalenz des rechtlichen und politischen Verhältnisses zwischen Kolonien und Metropole kommt in der „Schutz“-Semantik deutlich zum Ausdruck: „Der Begriff sollte getreu dem Motto, dass die Flagge dem Handel folgen müsse, dem Willen der Reichsregierung Ausdruck verleihen, lediglich den ‚Schutz‘, der sich in diesen Gebieten aufhaltenden Reichsangehörigen staatlich zu garantieren, nachdem bereits – in aller Regel auf private Initiative – Verträge mit Einheimischen über deren ‚Schutz‘ oder die Abtretung von Gebieten geschlossen worden waren“ (Nagl 2007, S. 24).

22 Somit war der Reichstag nicht für den Erlass von Gesetzen für die Kolonien zuständig. Die Kolonien wurden vom Kaiser, der über alle drei Gewalten (Legislative, Exekutive und Judikative) verfügte und diese an den jeweiligen Gouverneur delegierte, direkt durch Runderlasse regiert (Nagl 2007, S. 30). In den Kolonien galten außerdem unterschiedliche Gerichtsbarkeiten für „Europäer“ und „Eingeborene“. In vielen Rechtsfällen musste erst die Zuständigkeit der jeweiligen Gerichtsbarkeit „geprüft“ werden – was *de facto* in einer Klassifikation in die eine oder andere Kategorie (aufgrund von Religion, „Lebensführung“ und Staatsangehörigkeit) resultierte (Schaper 2012).

23 Die Rechtsauslegung des Auswanderungsgesetzes von 1897 für die Verwaltung definierte deutsche Kolonien als „nicht fremde“ Gebiete und schloss somit Passagiere auf dem Weg zu deutschen Kolonien aus der Kategorie „Auswanderer“ aus (Renard 2021).

24 Zuvor war das „Dezernat für die deutschen überseeischen Interessen“ der politischen Abteilung des Auswärtigen Amts zuständig.

25 Zur Kolonialreform und Politik Bernhard Dernburgs vergleiche Utermark (2012).

26 Im französischen Kontext war ebenfalls die Kolonialverwaltung für die Durchführung statistischer Beobachtungen verantwortlich. Vor dem Ersten Weltkrieg verfügte diese nicht über ausgebildete Mitarbeiter für statistische Operationen.

27 Zu den „Verträgen“ zwischen europäischen Kolonisierenden und afrikanischen (Staats‑)Oberhäuptern vgl. Surun (2014, 2019, S. 25).

28 Oftmals wurde die genaue Reichweite der Rechtsübertragung nicht näher spezifiziert; so verwischten die Grenzen zwischen dem privaten Landverkauf und einem angeblichen Souveränitätstransfer, vergleiche Coret (2019) und Surun (2014).

29 Für Achille Mbembe (2000, S. 265) lässt sich die Trennung zwischen Küste und Hinterland sowie die Herausbildung von Enklaven in vielen afrikanischen Ländern auf die Zeit der ersten Kontakte mit europäischen Händlern zurückführen. Laut Clélia Coret zeugt diese Raumordnung auch von der politischen Organisation an der ostafrikanischen Küste vor der Kolonialzeit, wo sich unabhängige Stadtstaaten als Inseln der Macht und Souveränität seit dem Mittelalter konstituiert hatten (Coret 2019, S. 53 ff.).

30 Gerardo Serra (2014) und James Duminy (2017) sprechen für die britischen Kolonien Gold Coast (heute Ghana) und Kenia von einer „uneven topography“ der Statistik.

31 Station in Deutsch-Ostafrika, heute Tansania.

32 Für Beispiele aus der preußischen Statistik vgl. Wobbe (2021).

33 Zur Materialität der statistischen Praxis in Preußen vgl. von Oertzen (2017).

34 Zuvor war die Handelsstatistik reformiert worden (Kol.-Abt. des Auswärtigen Amts 1903, S. 409).

35 Benno Gammerl (2010, S. 317 ff.) spricht von einem ähnlichen Prozess der „Spaltung der Bevölkerung“ auf der Ebene des Rechts im Fall des Britischen Empires.

36 Wie in der Metropole herrschte auch in den deutschen Kolonien Meldepflicht für die „europäische“ Bevölkerung. Als Standesbeamten führten Gouverneure und Bezirksamtmänner spätestens ab 1890 Melderegister über die anwesende „europäische“ Bevölkerung zu Ehen, Geburten und Todesfällen (Grosse 2000, S. 150).

37 Der Gouverneur der Kolonie Deutsch-Ostafrika spricht „zur Ergänzung der Personenstandsbücher“ von „eine[r] auf der Personalkenntnis seitens der einzelnen Beamten fußende[n] stillschweigende[n] Volkszählung“ (Schreiben, Gouverneur von Deutsch-Ostafrika, an Kol.-Abt., 09.10.^1902^).

38 Für die Kolonie Togo im Zeitraum von 1899–1913 hat Trutz von Trotha (1994, S. 397) ermittelt, dass 23 % der statistischen Daten auf Zählungen und 31 % auf Schätzungen zurückzuführen sind. Die restlichen 41 % wurden aus dem Vorjahr übernommen.

39 Andererseits wurden herausragende Individuen (z. B. muslimische Autoritäten in Französisch-Westafrika, vgl. Dramé 2017) namentlich benannt und als besonders „gefährlich“ eingestuft und überwacht.

40 Im preußischen Zensus galt das Individuum seit 1867 als Grundeinheit; dieses Prinzip wurde nach 1871 von der Reichstatistik übernommen (von Oertzen 2017, S. 415).

41 „Kapländer“ und „Buren“ werden in den Denkschriften durchgängig der „europäischen“ oder „weißen“ Bevölkerung zugerechnet, jedoch durch die Angabe der „Nationalität“ von den restlichen „Europäern“, insbesondere von den Deutschen, getrennt, um die parallele Entwicklung beider Gruppen zu vergleichen und die Besiedlungspolitik Deutsch-Südwestafrikas zu steuern (dazu vgl. Kundrus 2003, S. 96 ff.).

42 So wurden in der Kolonie Deutsch-Ostafrika bis zum Berichtsjahr 1899–1900 „Türken“, „Syrer“ und „Armenier“ als Teil der „europäischen Bevölkerung“ angesehen. In der darauffolgenden Denkschrift sind „Syrer“ und „Armenier“ der „nichteuropäischen Bevölkerung“ zugeordnet, mit der Begründung, dass sie (zusammen mit „Indern“, „Arabern“ und „Goanesen“) „in Bezug auf Rasse und Kultur eine Zwischenschicht zwischen den Europäern und den Eingeborenen des Schutzgebietes dar[stellen]“ (Denkschrift über das Berichtsjahr 1900/1901 (1902), S. 2914). Ab dem Berichtsjahr 1903–1904 wurden auch „Türken“ in der Kategorie „nicht eingeborene farbige Bevölkerung“ sortiert und somit aus der „weißen Bevölkerung“ ausgeschlossen.

43 Auch im Falle Britisch-Indiens wird der Relevanzgewinn „‚rassische[r]‘ Kategorien“ im Recht und in der Statistik erst um die Jahrhundertwende datiert (Gammerl 2010, S. 140 ff.).

44 Organisationen, die sich für die Rechte von Indigenen einsetzen, heben die postkolonialen Kontinuitäten dieses ungleichen Wissensregimes im Kontext von COVID-19 hervor: https://www.culturalsurvival.org/news/right-be-named-and-counted-indigenous-peoples-are-excluded-covid-19-statistics (Zugegriffen: 23. Okt. 2020).

45 So wurde argumentiert, dass sich Kolonisierte aus diesem Grund gegen die Zählung wehren würden – was auch in vielen Fällen geschah. Heute wird dies als strategischer Widerstand gegen die Kolonialherrschaft und die Steuererhebung interpretiert, die oft Anlass für Zählungen war (von Trotha 1994, S. 397).

46 Nichteuropäische Bevölkerungsgruppen als defizitär zu bezeichnen – im Vergleich zur angeblichen Modernität der Europäer –, war ein klassisches Muster der Ethnologie und insgesamt des Wissens über die „Anderen“ um 1900, wie am Beispiel der Unterscheidung zwischen „schriftlicher“ und „oraler“ Kultur deutlich wird (Schüttpelz 2005).

47 So die Denkschrift für das Berichtsjahr 1898/1899 (1899, S. 2726) über die Bewohner der Kolonie Deutsch-Südwestafrika: „Der mit der Vornahme eines zuverlässigen Census verbundene Zeit- und Kostenaufwand würde zu dem wirthschaftlichen Werth des Ergebnisses, zur Zeit noch in keinem Verhältnisse stehen“.

48 Zitiert in Luks (2019, S. 186–189).

49 Die Erforschung ökonomischer Verhältnisse in den Kolonien wurde der Ethnologie überlassen, die sich in der Zwischenkriegszeit als wichtige Stütze einer auf Effizienz gerichteten Kolonialpolitik profilieren konnte (Speich Chassé 2015, S. 597; vgl. auch Luks 2019, S. 34).

50 Der Begriff „situation coloniale“ wurde 1951 vom französischen Soziologen Georges Balandier geprägt. Für eine historische Kontextualisierung vgl. Speich Chassé (2016).

51 Hier sind die Mobilitätskontrolle und -verbote mittels sogenannter „Eingeborenen Passmarken“ in der Kolonie Deutsch-Südwestafrika ab 1907 zu nennen. Diese waren im Zuge des Völkermords Teil einer Reihe repressiver Maßnahmen (Internierung in Konzentrationslagern, Zwangsarbeit und Deportation) gegen die Herero und Nama. Die „Marke“ bestand aus einer Metallplatte, die mit einer Nummer graviert war und um den Hals getragen werden musste. Die persönlichen Nummern wurden in einem Register verzeichnet; für jede Reise außerhalb des Distrikts musste ein Reisepass beantragt werden. Dazu vgl. Krüger (1999, S. 137 ff.).

52 Dies wird auch an dem rechtlich ungeklärten Status der Kolonisierten im sogenannten „Schutzgebietsgesetz“ deutlich.

53 Bereits auf dem Kolonialkongress von 1905 in Berlin wurden Stimmen laut, die für die Einführung von Kursen über die Rechtsnormen und die Sprachen der Kolonisierten in die Ausbildung von Kolonialadministratoren argumentierten (Deutscher Kolonialkongress 1906).

## References

[CR1] Aguilera M (2017). L’ingénieur, les capitaines et les planteurs: Le recensement de la Siempre Fiel Isla de Cuba (1825–1842). Entre savoirs locaux et préoccupations impériales. Histoire & Mesure.

[CR2] Bayly CA (1996). Empire and Information. Intelligence Gathering and Social Communication in India, 1780–1870.

[CR3] Behrisch L (2006). Vermessen, Zählen, Berechnen. Die politische Ordnung des Raums im 18. Jahrhundert.

[CR4] Behrisch L (2016). Die Berechnung der Glückseligkeit. Statistik und Politik in Deutschland und Frankreich im späten Ancien Régime.

[CR5] Benton L (2009). A Search for Sovereignty. Law and Geography in European Empires, 1400–1900.

[CR6] Berg G, Török BZ, Twellmann M (2015). Berechnen, Beschreiben. Praktiken statistischen (Nicht‑)Wissens, 1750–1850.

[CR7] Brian É (1989). Y a-t-il un objet Congrès? Le cas du Congrès international de statistique (1853–1876). *Mil neuf cent*. Revue d’histoire intellectuelle (Cahiers Georges Sorel).

[CR8] Brian É (1994). La Mesure de l’État. Administrateurs et géomètres au XVIIIe siècle.

[CR9] Burbank J, Cooper F (2010). Empires in world history. Power and the politics of difference.

[CR10] Cohn BS, Cohn BS (1987). The census, social structure, and Objectification in south Asia. An anthropologist among the historians and other essays.

[CR11] Conrad S, Conrad S, Osterhammel J (2004). „Eingeborenenpolitik“ in Kolonie und Metropole. „Erziehung zur Arbeit“ in Ostafrika und Ostwestfalen. Das Kaiserreich transnational. Deutschland in der Welt 1871–1914.

[CR12] Conrad S, Osterhammel J (2004). Das Kaiserreich transnational. Deutschland in der Welt 1871–1914.

[CR13] Cooper F (2004). Development, modernization, and the social sciences in the era of decolonization. The examples of British and French Africa. Revue d’histoire des sciences humaines.

[CR14] Cordell DD, Ittmann K, Maddox GH, Ittmann KDDC, Maddox GH (2010). Counting subjects. Demography and empire. The demographics of empire: the colonial order and the creation of knowledge.

[CR15] Coret C (2019). La souveraineté de Witu au XIXe siècle. De la refondation à la colonisation d’une cité-État sur la côte est-africaine. Revue d’histoire du XIXe siècle.

[CR16] Crampton JW, Elden S (2007). Space, knowledge and power. Foucault and geography.

[CR17] Desrosières A (1997). Du territoire au laboratoire: la statistique au XIXe siècle. Courrier des statistiques.

[CR18] Desrosières A (2001). Entre réalisme métrologique et conventions d’équivalence. Les ambiguïtés de la sociologie quantitative. Genèses.

[CR19] Desrosières A, Touffut J-P (2007). Comparer l’incomparable : essai sur les usages sociaux des probabilités et des statistiques. La société du probable. Les mathématiques sociales après Augustin Cournot.

[CR20] Douglas M (1986). How institutions think.

[CR21] Dramé, Amadou. 2017. Que révèle le “formulaire d’enquête interne” d’El hadji Mamadou Kaba Diakité? https://ihacrepos.hypotheses.org/658 (Zugegriffen: 03. Dez. 2019).

[CR22] Duminy J (2017). A piecemeal avalanche. The uneven topography of statistics in colonial Kenya, c. 1900 to 1952. Urban Forum.

[CR23] Eckert A, Pesek M, Conrad S, Osterhammel J (2004). Bürokratische Ordnung und koloniale Praxis. Herrschaft und Verwaltung in Preußen und Afrika. Das Kaiserreich transnational. Deutschland in der Welt 1871–1914.

[CR30] Encarnación GR, Pieper M, Pieper M (2003). Einleitung. Gouvernementalität. Ein sozialwissenschaftliches Konzept in Anschluss an Foucault.

[CR24] Epple A, Erhart W (2015). Die Welt beobachten. Praktiken des Vergleichens.

[CR25] Foucault M (2004). Sécurité, territoire, population. Cours au Collège de France. 1977–1978.

[CR26] Gammerl B (2010). Untertanen, Staatsbürger und Andere. Der Umgang mit ethnischer Heterogenität im Britischen Weltreich und im Habsburgerreich 1867–1918.

[CR97] Gervais RR, Mandé I (2007). Comment compter les sujets de l’Empire? *Vingtième*. Siècle. Revue d’histoire.

[CR27] Go J, Burzan N (2019). Taking Empire Seriously. Komplexe Dynamiken globaler und lokaler Entwicklungen.

[CR28] Gräbel C (2015). Die Erforschung der Kolonien: Expeditionen und koloniale Wissenskultur deutscher Geographen, 1884–1919.

[CR29] Grosse P (2000). Kolonialismus, Eugenik Und Bürgerliche Gesellschaft in Deutschland 1850–1918.

[CR31] Hacking I (1982). Biopower and the avalanche of printed numbers. Humanities in Society.

[CR32] Harbach H (2012). Computer und menschliches Verhalten.

[CR33] Heintz B (2016). „Wir leben im Zeitalter der Vergleichung“. Perspektiven einer Soziologie des Vergleichs. Zeitschrift für Soziologie.

[CR98] Heintz B, Heintz B, Wobbe T (2021). *Big Observation* – Ein Vergleich moderner Beobachtungsformate am Beispiel von amtlicher Statistik und Recommendersystemen. Soziale Praktiken des Beobachtens: Vergleichen, Bewerten, Kategorisieren und Quantifizieren, Kölner Zeitschrift für Soziologie und Sozialpsychologie Sonderheft 73.

[CR34] Heintz B, Werron T (2011). Wie ist Globalisierung möglich? Zur Entstehung globaler Vergleichshorizonte am Beispiel von Wissenschaft und Sport. Kölner Zeitschrift für Soziologie und Sozialpsychologie.

[CR35] Hotten JC (1874). The original lists of persons of quality (…) who went from Great Britain to the American plantations 1600–1700.

[CR36] Isin E, Ruppert E, Didier Bigo, Isin E, Ruppert E (2019). Data’s empire. Postcolonial data politics. Data politics: worlds, subjects, rights.

[CR37] Ittmann KDDC, Maddox GH (2010). The demographics of empire. The colonial order and the creation of knowledge.

[CR38] Joyce P, Joyce P (2002). Maps, blood and the city. The governance of the social in nineteenth-century Britain. The Social in Question. New bearings in history and the social sciences.

[CR39] Jureit U (2015). Herrschaft im Kolonialen Raum. Territorialität als Ordnungsprinzip. Aus Politik und Zeitgeschichte.

[CR40] Jureit U (2016). Das Ordnen von Räumen. Territorium und Lebensraum im 19. und 20. Jahrhundert.

[CR41] Kalpagam U (2014). Rule by numbers. Governmentality in colonial India.

[CR42] Kateb K (2004). La statistique coloniale en Algérie (1830–1962). Courrier des statistiques.

[CR43] Krüger G (1999). Kriegsbewältigung und Geschichtsbewusstsein. Realität, Deutung und Verarbeitung des deutschen Kolonialkriegs in Namibia 1904 bis 1907.

[CR44] Kundrus B (2003). Moderne Imperialisten. Das Kaiserreich im Spiegel seiner Kolonien.

[CR45] Legg S, Crampton JW, Elden S (2007). Beyond the European Province. Foucault and Postcolonialism. Space, Knowledge and Power. Foucault and Geography.

[CR46] Leonhard J, von Hirschhausen U (2011). Empires und Nationalstaaten im 19. Jahrhundert.

[CR47] Luks T (2019). Die Ökonomie der Anderen.

[CR48] Luste Boulbina S (2006). Les colonies. Une réalité fantôme. Les Temps Modernes.

[CR49] Mbembe A (2000). At the edge of the world. Boundaries, territoriality, and sovereignty in africa. Public Culture.

[CR50] Nagl D (2007). Grenzfälle. Staatsangehörigkeit, Rassismus und nationale Identität unter deutscher Kolonialherrschaft.

[CR51] von Oertzen C (2017). Die Historizität der Verdatung. Konzepte, Werkzeuge und Praktiken im 19. Jahrhundert. N.T.M. Zeitschrift für Geschichte der Wissenschaften, Technik und Medizin.

[CR52] Pesek M (2005). Koloniale Herrschaft in Deutsch-Ostafrika. Expeditionen, Militär und Verwaltung seit 1880.

[CR53] Pieper M, Atzert T, Karakayali S, Tsianos V (2011). Biopolitik – in der Debatte.

[CR54] Porter TM (1986). The rise of statistical thinking: 1820–1900.

[CR55] Randeraad N (2010). States and statistics in the nineteenth century. Europe by numbers.

[CR56] Rapior R, Bennani H, Bühler M, Cramer S, Glauser A (2020). Bringing the Empire (Back) In: Zur Überwindung des Eurozentrismus in der Weltgesellschaftsforschung. Global beobachten und vergleichen. Soziologische Analysen zur Weltgesellschaft.

[CR57] Rassem MH (1980). Statistik und Staatsbeschreibung in der Neuzeit: vornehmlich im 16.–18. Jahrhundert.

[CR58] Renard L (2021). The statistical construction of otherness. Colonial, national and migratory classification principles in France and Germany (1880–2010).

[CR59] Renard L, Wobbe T (2019). La statistique internationale comme instrument de globalisation? La carrière de la catégorie « travailleurs familiaux » au sein de l’Organisation internationale du travail (1919–1982). Revue française de sociologie.

[CR60] Santos CM (2010). Administrative knowledge in a colonial context. Angola in the eighteenth century. The British Journal for the History of Science.

[CR61] Schaper U (2012). Koloniale Verhandlungen. Gerichtsbarkeit, Verwaltung und Herrschaft in Kamerun 1884–1916.

[CR62] Schlögel K (2006). Im Raume lesen wir die Zeit. Über Zivilisationsgeschichte und Geopolitik.

[CR63] Schüttpelz E (2005). Die Moderne im Spiegel des Primitiven. Weltliteratur und Ethnologie (1870–1960).

[CR64] Scott D (1995). Colonial governmentality. Social Text.

[CR65] Serra G (2014). An uneven statistical topography: the political economy of household budget surveys in late colonial Ghana, 1951–1957. Canadian Journal of Development Studies.

[CR66] Speich Chassé D (2015). Die „Dritte Welt“ als Theorieeffekt. Ökonomisches Wissen und globale Differenz. Geschichte und Gesellschaft.

[CR67] Speich Chassé, Daniel. 2016. La situation coloniale. http://livingbooksabouthistory.ch/en/book/la-situation-coloniale (Zugegriffen: 15. Okt. 2020).

[CR68] Speich Chassé D, Bennani H, Bühler M, Cramer S, Glauser A (2020). Der Staatsvergleich in historischer Perspektive: Warum, seit wann und wie werden politische Mächte miteinander verglichen?. Global beobachten und vergleichen. Soziologische Analysen zur Weltgesellschaft.

[CR69] Steinmetz G (2007). The devil’s handwriting. Precoloniality and the German colonial state in Qingdao, Samoa, and Southwest Africa.

[CR70] Steinmetz G (2008). The colonial state as a social field. Ethnographic capital and native policy in the German overseas empire before 1914. American Sociological Review.

[CR71] Steinmetz G (2017). Sociology and colonialism in the British and French empires, 1945–1965. The Journal of Modern History.

[CR72] Steinmetz W (2019). The force of comparison. A new perspective on modern European history and the contemporary world.

[CR100] Steinmetz W, Heintz B, Wobbe T (2021). Macht – Leistung – Kultur: Staatenvergleiche vom 17. bis ins frühe 20. Jahrhundert. Soziale Praktiken des Beobachtens: Vergleichen, Bewerten, Kategorisieren und Quantifizieren, Kölner Zeitschrift für Soziologie und Sozialpsychologie Sonderheft 73.

[CR73] Stoler AL (1995). Race and the education of desire. Foucault’s history of sexuality and the colonial order of things.

[CR74] Stoler AL (2009). Along the archival grain. Epistemic anxieties and colonial common sense.

[CR75] Surun I (2014). Une souveraineté à l’encre sympathique ? Souveraineté autochtone et appropriations territoriales dans les traités franco-africains au XIXe siècle. Annales. Histoire, Sciences Sociales.

[CR76] Surun I (2019). Introduction. Trajectoires historiques des souverainetés africaines au XIXe siècle. Revue d’histoire du XIXe siècle.

[CR78] Touchelay B, Fichter JR (2019). British and French colonial statistics. Development by hybridization from the nineteenth to the mid-twentieth centuries. British and French colonialism in Africa, Asia and the Middle East. Connected empires across the eighteenth to the twentieth centuries.

[CR77] von Trotha T (1994). Koloniale Herrschaft. Zur soziologischen Theorie der Staatsentstehung am Beispiel des „Schutzgebietes Togo“.

[CR79] Utermark S (2012). „Schwarzer Untertan versus schwarzer Bruder.“ Bernhard Dernburgs Reformen in den Kolonien Deutsch-Ostafrika, Deutsch-Südwestafrika, Togo und Kamerun.

[CR80] Wells RV (2015). Population of the British colonies in America before 1776. A survey of census data.

[CR81] Wimmer A, Min B (2006). From empire to nation-state. Explaining wars in the modern world, 1816–2001. American Sociological Review.

[CR99] Wobbe T, Heintz B, Wobbe T (2021). Die Differenz Haushalt vs. Markt als latentes Beobachtungsschema. Vergleichsverfahren der inter/nationalen Statistik (1882–1990). Soziale Praktiken des Beobachtens: Vergleichen, Bewerten, Kategorisieren und Quantifizieren, Kölner Zeitschrift für Soziologie und Sozialpsychologie Sonderheft 73.

[CR82] Zerubavel E (1996). Lumping and splitting. Notes on social classification. Sociological Forum.

[CR85] Denkschrift über das ostafrikanische und über das südwestafrikanische Schutzgebiet, Reichstagsprotokolle, Aktenstück Nr. 48, 1893/1894, S. 328–357. (Am 17.11.1893 dem Reichstag übermittelt).

[CR86] Denkschrift über die Entwicklung der Deutschen Schutzgebiete in Afrika und der Südsee mit Ausnahme von Deutsch-Ostafrika im Jahre 1898/1899, Reichstagsprotokolle, Aktenstück Nr. 508, 1898/1900, S. 2667–2765 (Am 02.12.1899 dem Reichstag übermittelt).

[CR87] Denkschrift über die Entwicklung der deutschen Schutzgebiete in Afrika und der Südsee, Berichtsjahr 1900/1901, Reichstagsprotokolle, Aktenstück Nr. 437, 1900/1903, S. 2907–3158. (Am 29.01.1902 dem Reichstag übermittelt).

[CR95] Deutscher Kolonialkongress (1906). Verhandlungen des Deutschen Kolonialkongresses 1905 zu Berlin am 5., 6. und 7. Oktober 1905.

[CR84] Direktor der Kol.-Abt. (Kayser), Runderlass, 4.05.1891, BArch R 1001/6466 (Allgemeine Jahresberichte aus Deutsch-Ostafrika, Bd. 1, Januar 1894–29. November 1896).

[CR92] Gouverneur von Deutsch-Ostafrika an Kol.-Abt., 9.10.1902, BArch R1001/7428 (Bevölkerungsstatistik für Deutsch-Ostafrika).

[CR94] Great Britain. Census Office (1906). Census of the British empire. 1901.

[CR88] Hermann R (1901). Statistik der fremden Bevölkerung in den deutschen Schutzgebieten.

[CR89] Hermann R, Zahn F (1911). Kolonialstatistik. Die Statistik in Deutschland nach ihrem heutigen Stand.

[CR90] Kol.-Abt. (an Gouverneure?), Schreiben, 1901, BArch R1001/7427 (Bevölkerungsstatistik der deutschen Kolonien, Allgemeines).

[CR91] Kol.-Abt. an Gouverneure, Runderlass über Bevölkerungsstatistiken, 8.01.1902, BArch R 1001/6673 (Runderlasse der Kolonialabteilung, Bd. 1, Januar 1901–September 1903).

[CR93] Kolonial-Abteilung des Auswärtigen Amts, 15.08.1903. Die Neuordnung der kolonialen Bevölkerungsstatistik. *Deutsches Kolonialblatt* 14(16):409–414.

[CR83] Quételet A (1873). Congrès international de statistique. Sessions de Bruxelles (1853), Paris (1855), Vienne (1857), Londres (1860), Berlin (1863), Florence (1867), La Haye (1869) et St-Pétersbourg (1872).

[CR96] Reichskolonialamt, Entwurf eines Runderlasses, 3.07.1907, BArch R 1001/6460 (Jahresberichte allgemeinen Inhalts aus den Kolonien, Bd. 5, März 1906–März 1918).

